# Accessing primary care and the importance of ‘human fit’: a qualitative participatory case study

**DOI:** 10.3399/BJGP.2021.0375

**Published:** 2022-02-08

**Authors:** Jennifer Voorhees, Simon Bailey, Heather Waterman, Kath Checkland

**Affiliations:** National Institute for Health Research clinical lecturer;; Centre for Health Services Studies, University of Kent, Kent.; Cardiff University, Cardiff.; Centre for Primary Care and Health Services Research, University of Manchester, Manchester.

**Keywords:** access, general practice, population health, service organisation, inequalities, continuity of care

## Abstract

**Background:**

Good access to primary care is an important determinant of population health. While the academic literature on access to care emphasises its complexity, policies aimed at improving access to general practice in the UK have tended to focus on measurable aspects, such as timeliness or number of appointments.

**Aim:**

To fill the gap between the complex understanding of primary care access in the literature and the narrow definition of access assumed in UK policies.

**Design and setting:**

Qualitative, community-based participatory case study within the geographic footprint of a clinical commissioning group in the north west of England. Data collection took place from October 2015 to October 2016. Purposive sampling and snowball approaches were used to achieve maximum variation among service users and providers across general practice settings.

**Method:**

Levesque *et al’s* conceptual framework of patient-centred access was applied and the study used multiple qualitative methods (interviews, focus groups, and observation). Analysis was ongoing, iterative, inductive, and abductive with the theory.

**Results:**

The comprehensiveness of Levesque *et al’s* access theory resonated with diverse participant experiences. However, while its strength was to highlight the importance of people’s abilities to access care, this study’s data suggest equal importance of healthcare workforce abilities to make care accessible. Thus, the authors present a definition of access as the ‘human fit’ between *the needs and abilities of people in the population and the abilities and capacity of people in the healthcare workforce*, and provide a modified conceptual framework reflecting these insights.

**Conclusion:**

An understanding of access as ‘human fit’ has the potential to address longstanding problems of access within general practice, focusing attention on the need for staff training and support, and emphasising the importance of continuity of care.

## INTRODUCTION

The ability of citizens to access the health care they need is one of the most important attributes of any health system, with access to primary care particularly important for addressing population health inequalities.^1^ This study applies an existing conceptual framework for understanding access to primary care and develops it further, drawing conclusions relevant to contemporary policy approaches to this important issue.

### Access theory: capturing complexity

International scholars studying healthcare access have long recognised a disparity between the simplistic approaches to access evident in policy and the complexities of how individuals actually access care,^2^ with policies often focused on timeliness or numbers of appointments, and tending to see continuity of care as something to be traded off against better access. Access theories, by contrast, aim to capture the complexity of individuals’ needs alongside characteristics of health systems and populations, issues which are often missing from policy. Donabedian identified ‘accessibility’ as *‘characteristics of the service that facilitate or obstruct use by potential clients.’* ^3^ Penchansky and Thomas elaborated on this, defining access *‘as a concept representing the degree of fit between clients and the system.’* ^4^ Aday and Andersen’s multiple models of access derived from patterns of utilisation, incorporating patients’ *‘predisposing, enabling, and need components’* as well as differentiating ‘potential’ from ‘realised’ access.^2^^,^^5^^–^^7^ More recent conceptual work has embraced these theories, defining access as *‘… a multidimensional concept based on the interaction (or degree of fit) between health care systems and individuals, households, and communities.’* ^8^

Qualitative research about patient experiences has defined additional concepts such as candidacy, which describes *‘the ways in which people’s eligibility for medical attention and intervention is jointly negotiated between individuals and health services.’* ^9^ Related work exploring access for people with mental health problems in the UK applied candidacy, alongside concordance (*‘the match between users’ and practitioners’ narratives and resources for successful access’*), and recursivity (*‘the interdependency of users’ experiences of health services and future actions in regards to health and help-seeking’*) to capture the nuances of people’s lived experiences.^10^ Importantly, in these patient-centred approaches, continuity of care is seen as a potential contributor to better access.

**Figure 1.**
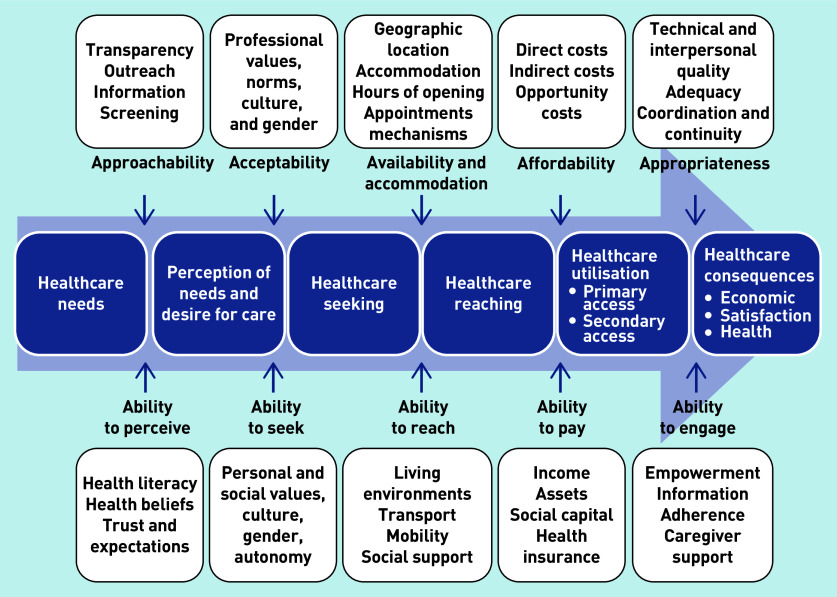
***A conceptual framework for patient-centred access to health care (reproduced from Levesque et al, 2013)*** *^11^*

In 2013, Levesque *et al*, synthesized a conceptual framework for *‘patient-centred access to health care’* (Figure 1),^11^ which juxtaposes five dimensions of accessibility of the healthcare system (approachability, acceptability, availability and accommodation, affordability, and appropriateness) with the abilities of patients to identify health needs, seek, reach, and utilise care.

**Table:** How this fits in

Access to general practice is an important topic that receives much attention. While research literature recognises access issues as complex, policy on access to general practice in the UK tends to define it narrowly, with unintended consequences. This study found that an existing comprehensive model of access resonated with frontline experiences. The study offers further modifications to the theory to make it practically useful for both policymakers and clinicians to address longstanding issues of access to general practice.

This conceptualisation builds on previous access literature and has multiple strengths, including conceptualising continuity as a component of access (within the dimension of appropriateness). While Levesque *et al* ’s definition of access does not include the concept of ‘fit’, their framework clearly depicts this dynamic, resonating with previous theories of access and concepts such as candidacy described above. For these reasons, this conceptualisation of access was applied within this research.

### UK access policy: narrow conceptualisation with unintended consequences

Despite the complexity evident in empirical studies of service access and manifest in associated theories, policies addressing access to general practice in the UK often assume a narrow definition of access, linked to measurable targets such as timeliness or number of appointments.^12^^,^^13^ This narrow approach fails to embrace the real-life complexity captured in theoretical literature, and has potentially worsened health inequalities and inequities of access to care.^14^ Importantly, rather than embracing continuity of care as a component of access, policy casts continuity as something separate, to be traded off against better access. Important policy examples include the focus on providing access within 48 hours in the 2000s, and the widespread adoption of ‘advanced access’ systems to meet those targets.^15^

Such policy solutions were adopted even though available evidence demonstrated that timeliness was not more important to patients or doctors than continuity.^16^^–^^18^ In England in the mid-2010s, policies to provide 7-day extended access to general practice services dominated. These were costly, not based on any recognised theory of access, and rolled out without any evidence that they were needed, further undermining continuity.^19^^–^^21^ Current access policy is, in part, driven by the 2019 Conservative election manifesto promise of 50 million more appointments a year in general practice.^22^ While superficially attractive as a simple and easily understood policy, this approach again prioritises volume of appointments over appropriateness or need.

This research therefore aimed to fill the gap between the complex understanding of access in the literature and the narrow definition of access assumed in many policies. The study explored how access theory, as depicted by Levesque *et al* ’s conceptual framework of access to care,^11^ resonated with a broad range of stakeholders, and considered how the theory could be developed to inform policy.

## METHOD

This was a qualitative participatory case study^23^ in the north west of England, using the Levesque *et al* theory^11^ as a tool within data collection and analysis. The study was based in the geographical area covered by the Tameside and Glossop (T&G) Clinical Commissioning Group (CCG). CCGs have delegated responsibility for overseeing primary care provision, commissioning care, and managing contracts, including implementing national access policies. Situating the study in a single CCG area allowed for in-depth exploration of the contextual factors affecting access to care in a mixed area covering both urban and rural communities.

### Community-based participatory research

This research adopted an approach derived from community-based participatory research.^24^^,^^25^ Building on initial contacts made with the CCG and relevant groups, such as Healthwatch, a community-based participatory research team was formed consisting of 12 members of the T&G community, including patients, carers, GPs, practice staff, commissioners, and the voluntary sector. Levesque *et al* ’s conceptual framework was used^11^ to introduce the community research team to the complexities of access to care. The community research team contributed to project design and to data collection and analysis, meeting 35 times over 4.5 years. The community research team represented a wide range of backgrounds and professional roles, and this diversity provided an important resource for the project.

### Data collection

Data collection took place from October 2015 to October 2016, and included 19 semi-structured interviews,^26^ 7 focus groups,^27^ and 71 hours of observation,^28^^,^^29^ (summarised in Table 1). Purposive sampling^27^ and snowball approaches^30^^,^^31^ were used to achieve maximum variation among service users and providers across general practice settings. Community research team members contributed to purposive sampling decisions. Interviews and focus groups explored participants’ experiences of accessing or providing care. Community research team members assisted with focus group facilitation. Non-participant observation in communal general practice spaces focused on receptionist activities and interactions with service users, and non-participant observation in relevant public meetings, observing how access was discussed by service users, providers, and commissioners. Levesque *et al* ’s conceptual framework of access to care^11^ was utilised as a visual prompt^26^ to support discussion during data collection in interviews and focus groups, and occasionally during observation sessions.

**Table 1.** Summary of interview, focus group, and observation data generated in this study

**Method**	**Events, *n***	**Participants, *n***	**Approximate hours**
Interviews: service users	9	9	12
Interviews: service providers	10	10 (3 also patients in area)	12
Focus groups: service users	6	30	10
Focus groups: service providers	1	5	1.5
Observation: surgeries	13	8 sites (including interactions with approximately 40 receptionists, GPs, practice managers, practice nurses, administrative staff, and patients)	45
Observation: meetings and events	12	Approximately 70 individuals across all meetings/events	26
Total	51	54 (+ approximately 110)	106.5

The 54 interview and focus group participants included service users (patients, carers, and voluntary sector roles) and service providers (GPs, practice managers, receptionists, commissioners, and other relevant NHS roles). The 39 service user participants included: people possessing each of the nine protected characteristics under the UK Equality Act 2010^32^ (age, disability, gender reassignment, marriage and civil partnership, race, religion or belief, sex, and sexual orientation), as well as patients with various health needs (both physical and mental); carers of people with chronic diseases, including dementia, mental health problems, and learning disabilities; people facing economic deprivation; people for whom English was not their first language; and members of relevant voluntary organisations. Patient and carer participants ranged in age from 26–79 years, with carers discussing patients ranging from age zero to 101 years. The 15 service provider participants had between four and 26 years of experience. The surgery sites included small and large surgeries in all five neighbourhoods of T&G, and the 7-day access hub sites. The meetings and events observed included CCG Governing Body and Primary Care Joint Committee meetings, and CCG and Healthwatch public events. In all, the study collected data that spanned experiences across approximately 36 of the 45 general practice/hub sites in T&G.

### Data analysis

An ongoing, iterative analysis process was influenced by grounded theory,^33^ the five-step framework approach,^34^^,^^35^ and abductive analysis,^36^ balancing inductive insights from the data with insights from theory. The community led the analysis, with community research team and academic research team members contributing to each of the five stages: familiarisation, building the coding tree, coding, charting, and mapping and interpretation. ‘Familiarisation’ included immersion in the raw data. Interviews and focus groups were recorded and independently transcribed, checked for accuracy, and anonymised by the research team before sharing with community team members. Handwritten fieldnotes were made during observation sessions and then expanded in typed form shortly after the sessions. ‘Building the coding tree’ included discussions with team members about memos and notes made during familiarisation, notes made during interviews on printouts of the Levesque *et al* model, and important emerging concepts. These discussions directed further sampling until it was agreed saturation^37^^,^^38^ was met when it was felt a diverse range of experiences across of a variety of participants and practice sites was understood. ‘Coding’ of the data using NVivo (version 11) was informed by the rich discussions with team members during data collection and analysis. Coded transcripts were discussed further with team members to ensure consistency with early discussions and their insights. ‘Charting’ consisted of arranging data by code and further analysing content within each code^39^ through continued discussion with team members. ‘Mapping and interpretation’ involved integration of insights, including advancing the access theory, building on the previous analysis stages.

## RESULTS

The study found that Levesque *et al* ’s^11^ depiction of access as a complex interaction between individuals seeking care and the structural and cultural characteristics of the healthcare-providing organisation was intuitively understood by both patients and care providers, and in observations the study was able to recognise elements of the framework as they occurred. However, it was found that the framework, as depicted by Levesque *et al*, failed to explain all of the features of the situations that were observed or heard described. This led to modification of the framework, and offering an incremental advancement of Levesque *et al* ’s original theory. The new formulation builds on the definition implicit in Levesque *et al* ’s model (access as a ‘fit’ between human needs and capabilities and the structures and organisational processes in healthcare organisations), but puts additional emphasis on the human interactions facilitating (or hindering) access, highlighting the fact that organisational structures and processes are mediated by individuals with different capabilities and with varying capacity to engage.

**Figure 2.**
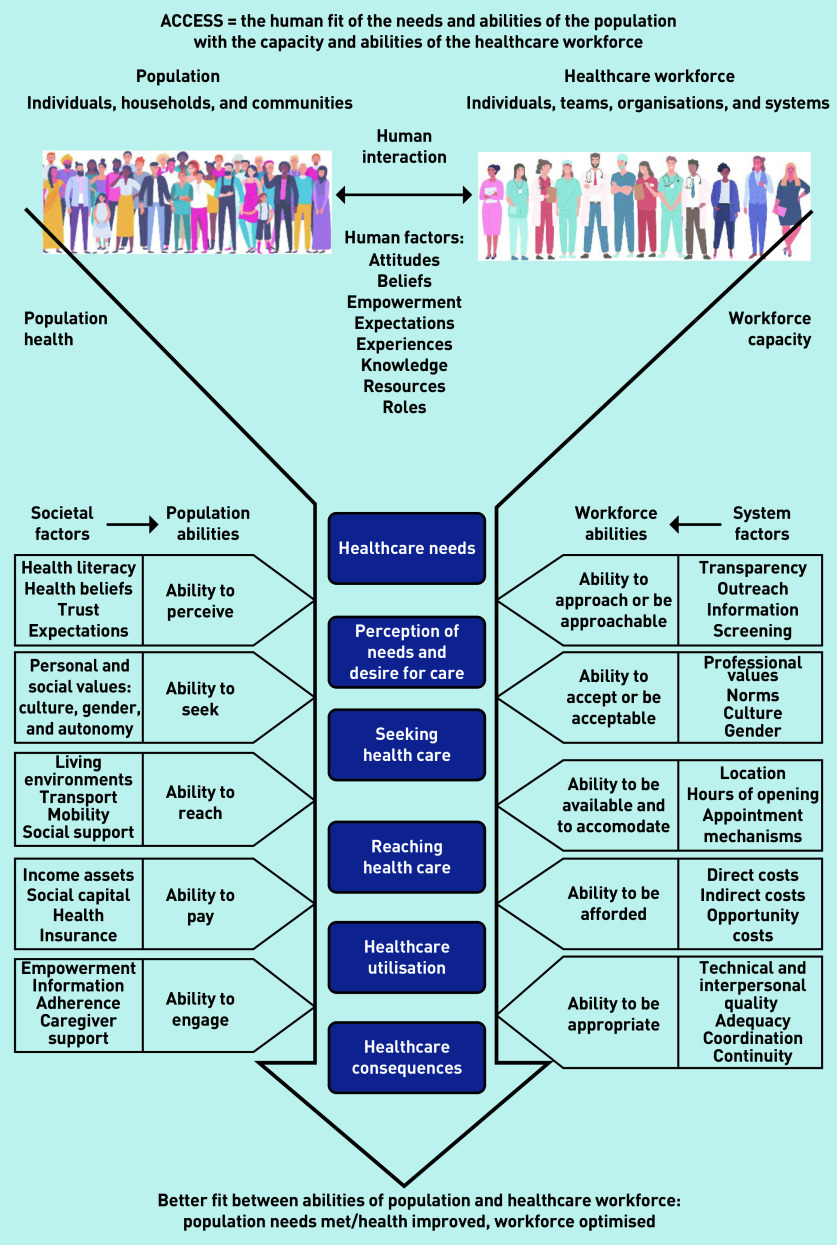
***Access as human fit (adapted from Levesque et al ’s conceptual framework for access to health care)*** *^11^*

This research therefore presents a definition of access as the ‘human fit’ of the *needs and abilities of the population* with the *capacity and abilities of the healthcare workforce*, in the context of particular societal conditions and organisational structures and processes. The modified theory is depicted in Figure 2. This new formulation retains many of Levesque *et al* ’s categories, but does two additional things: first, it emphasises that *human abilities* are important on both sides of the access equation, with those of the healthcare workforce as important as those of the population; second, it shows that both societal factors (affecting the population) and system factors (constraining staff) are mediated by these human abilities, and that both operate within a wider context characterised by the state of population health and the availability of healthcare workers. Furthermore, it illustrates how continuity matters throughout the human interactions at all stages of accessing care, not just within the dimension of appropriateness. Below, study data are presented, which support these modifications to the conceptualisation of access. Each responder has a unique code in which IR denotes ‘interview responder’ and FG denotes ‘focus group’ with individual responders.

### Stakeholder perceptions of access

Study participants (both service users and service providers) were enthusiastic about the complex depiction of access in the applied theory.^11^ This conceptualisation of access chimed with their experiences in a way that contemporary policy discourses did not.

For example, following a full discussion of experiences of accessing care, a patient participant viewing the visual prompt commented:

*‘It reflects, I think, the discussions we’ve just had, and it … fits with the way I’ve been trying to describe things, because I can recognise all of those, and I’ve alluded to them.’*

(IR01, patient)

A GP interview participant responded similarly:

Jennifer Voorhees (JV), interviewer: *‘How do you feel this idea of access resonates with you?’*

IR04, GP: *‘Really strongly. I think it’s very, very useful and tells you — it has just so much more in it than how people are usually seeing access.’*

JV: *‘You mentioned that this isn’t necessarily the way access is usually talked about.’*

IR04, GP: *‘No, it’s all about whether you can get an appointment within a certain length of time.’*

Participants offered additional insights and critiques based on their experiences. For example, an NHS staff member with public engagement experience appreciated the depiction of ‘abilities of people’ on the population side, but offered an insight about the importance of abilities of the healthcare workforce:

*‘I think it’s relevant ... Especially things like this: “the ability to reach, the ability to engage” … “the abilities of people” … I think that’s really, really, really important … because I don’t think they think about that enough … Also — and I don’t know whether it should be on here … there’s the abilities of GPs to be able to engage ... I think that is massively important … ’*

(IR10, NHS staff member)

This participant insight was one of several about the importance of the abilities of people within the workforce, and is explored further in the following section.

### Human fit: people and their abilities in context and the importance of continuity

Patients told us that they wanted to be treated as individuals, that they appreciated when receptionists knew them, and that this aspect of care had deteriorated compared with their experiences in the past. One commissioner explained how during engagement activities with members of the community around aspects of general practice, the top patient priority was to be treated as an individual, and that this was contrary to the current policy emphasis on timeliness of appointments:

*‘When I read back what people said and summarised it … people put at the top that they really liked being treated as an individual first. That was their ultimate priority … Appointments was important, but people didn’t sit there and all say “I can’t get an appointment.” They thought the topic needed looking at, but they had mixed experiences.’*

(IR07, commissioner)

As depicted in Figure 2, the modified theory reflects that feeling as if you are being treated as an individual relates to the receptionists’ and other care team members’ *abilities* to be approached, to accept, to accommodate, and to be appropriate, as well as their knowledge of particular individuals. Furthermore, it is not just the receptionist or clinician’s innate or learned abilities, but their capacity to use those abilities within the context of available resources, system factors, and workload pressures. A voluntary sector worker and patient reflected on how clinicians’ innate abilities were constrained by their workload:

*‘… some GPs can be challenging because of their own stress levels or their workload … In one surgery I’ll have a GP who is … the kind of person that would talk like you and I talk, and then say, “Is there anything else that I can help you with?” and, “How are you?” and will look you in the eye and will go away from their computer screen, and that’s how you want them to be. And with others, they will sit down, and they’ll say … “What do you want?” … and that’s not helpful … If you were somebody who wasn’t feeling very comfortable or wasn’t feeling very well or wasn’t feeling very in the mood to have a debate … you’d feel, “Well actually I’ll just take the prescription, thanks.”’*

(IR15 patient/voluntary sector worker)

To treat someone as an individual requires some knowledge of them or at least openness to that knowledge. This was observed to occur more readily in smaller practices. For example, in small practices receptionists often provided significant continuity for the patients, knowing them and their circumstances well. Some GPs recognised the role receptionists play in knowing patients within the practice population:

*‘And they also build up a knowledge of the individual characters who are ringing us up and how to handle them. So yeah, they’re on the same journey as us, in that respect.’*

(IR13, GP)

Participants recognised that continuity was important across the clinical team:

*‘Yeah … going back to this team and longevity in the practice, having a stable team of somebody saying, “I always go to* [name] *, the healthcare assistant. He’s great. I’ve been going for five years.”’*

(IR07, commissioner)

It was noticeable that, while patients requested clinicians who were known to them, many practice appointment systems were unable to accommodate this, leading to conflicts. An exchange from a focus group of practice managers demonstrates workload and doctor turnover limiting capacity to offer continuity:

*‘We had an example just this morning, a patient came in, and admittedly she’d come down because she said she couldn’t get through on the phone … She’s known to us, quite bad asthmatic, she was having a flare-up of her asthma, but only wanted to see one of two particular GPs. One was actually off on long-term sick, don’t know when he’s coming back, the other one was fully booked, but we had an appointment with a locum and another GP. We could have given her an appointment within 40 minutes of her turning up at the window, and it wasn’t good enough for her … She went away saying that she was going to complain. Now to me, if you’re really ill … you’ll see anybody who’s qualified to see you, and you don’t refuse an appointment within 40 minutes. I actually think that that’s quite good to be offered something so quick, but it wasn’t good enough, and I’m fully expecting, when I’m back in … that she’ll have written in and complained, and this is the kind of thing that you’re up against all the time.’*

(FG1R2, practice manager)

Thus, the data demonstrates how continuity can be an important component of good access, but emphasises that it is also a complex construct. In particular, the human skills of all members of the practice team are important in determining whether or not patients feel ‘known’ and therefore understood. Moreover, the current context of general practice with high workload and shortages of staff requires patients to also be adaptable and to show understanding of the pressures being experienced by staff.

### Improving access: relevance of the concept of ‘human fit’

In addition to illuminating current access issues as experienced by patients and staff, applying the new conception of access as the human fit between the abilities of both patients and staff can help to define ‘better’ access, providing specific targets for change. For example, the data suggest that improving access could mean improving continuity, providing more opportunity to achieve that human fit between staff and patients. Participants felt that the access theory was useful for this purpose as well:

*‘What you can almost come out with is a toolkit to help you in your area look at access seriously. So this could be* [pointing to diagram] *, these could be the ways you could start to look at access. Not “the answer”, it’s, “Before you look at that, think about this in your patch.”’*

(IR07, commissioner)

The complexity of human factors affecting access and the need for a proactive outreach to patients with unmet needs were relevant issues that participants felt were not appreciated by politicians. This GP explained:

*‘I don’t think the politicians appreciate any of that … They just see it as: if you’ve … got the flu, how quickly can you get to see your GP? Not that you need to see your GP with the flu, but that’s their own direct experience of doctors, and they don’t give any thought to the opportunistic stuff, the preventative stuff, the vulnerable people who don’t actually ask for appointments, but need probably more care than most voting adults. And so yeah, there is just an impoverished debate around it. There’s a lack of imagination about the true nature of the problem.’*

(IR13, GP)

Some GPs recognised that understanding access in this way and rethinking some of the rules within general practice around appointments were key to addressing health inequalities:

*‘There should never be a one-size-fits-all rule, should there? I think flexibility is definitely the route to helping with health inequalities.’*

(IR04, GP)

This research suggests that applying the ‘access as human fit’ conceptualisation has the potential to stimulate the imagination, highlight the breadth of issues to be addressed, and broaden targets for change. For example, by moving beyond timeliness to consider the human abilities and factors affecting interactions between the population and healthcare staff, as depicted in Figure 2.

## DISCUSSION

### Summary

This study demonstrates the relevance of a broad and complex theory of access, and advances a modified concept of access as the ‘human fit’ between the needs and abilities of the population with the capacity and abilities of the healthcare workforce. This modified concept of access, as depicted in Figure 2, builds on Levesque *et al* ’s patient-centred access concept^11^ to highlight the importance of both human and contextual factors within society and the healthcare system. Through qualitative and participatory research, this study demonstrates diverse service user and workforce stakeholder perceptions of accessing primary care in the UK. The relevance of continuity as a component of access, rather than something separate, has been emphasised. The multiple human and contextual factors of access, as depicted by the model in Figure 2, create opportunities to address components of access that have been overshadowed by historical policy emphasis on timeliness or number of appointments. The data presented demonstrate that attention to these other factors would be welcomed by both service users and the healthcare workforce, who have long recognised their value and contribution to longstanding access problems.

### Strengths and limitations

The study’s main strength lies in the application and advancement of a theoretical framework.^40^ Triangulation of methods provided a detailed understanding from multiple perspectives,^41^ and the participatory approach ensured that the research was grounded in and steered by the lived experiences of the local community.

A potential limitation is that the study occurred in one area. However, the ability to forge partnerships in the community and to explore the area in depth supported successful recruitment, including from groups sometimes termed ‘hard to reach’. A further limitation is the potential for social desirability bias, in that participants could respond positively to the access theory in order to please the researcher. However, participants were encouraged to tell their own story before mentioning the theory, and efforts were made to encourage critique.

### Comparison with existing literature

This work resonates with existing theoretical literature on access to health care, as well as with previous calls for access theory to be applied within service improvement and research.^14^^,^^42^ It resonates with other theories such as candidacy that embrace a recursive and dynamic view of access interactions.^9^ While Levesque *et al* ’s original theory includes aspects of the human capabilities of service providers under the heading of ‘appropriateness’, the study suggests that these capabilities are important across all of the dimensions of access. Thus, for example, ‘ability to be approachable’ highlights that the extent to which a service is experienced as approachable will be determined by *both* the design of the system *and* the human abilities of those greeting service users. This study offers an incremental adjustment of the original theory, which is a novel contribution.

### Implications for research and practice

The concept of access as human fit can be applied to inform the design of interventions to improve access and evaluations of access policies and interventions. Further research is also needed with practices and primary care networks to determine how to best apply this understanding of access in practical ways to improve fit for those in the population with unmet needs. Such research should continue to take a participatory approach in order to ensure patient and public perspectives shape the research efforts.

Politicians and policymakers can apply this understanding of access to convey a different goal for improving access to care: better human fit. Policies that take account of this conceptualisation would see continuity as an important contributor to better access, with attendant improvements in a wide range of outcomes (including mortality,^43^ accident and emergency use,^44^ hospital admissions,^45^ and patient and provider satisfaction,^17^^,^^18^) rather than as something that must be traded away for improved speed.

Wider application of this conceptualisation may support commissioners and providers to work in partnership with patients to improve access as human fit. Interventions are often aimed at the health service side, including creating more services,^46^ but this framework supports focusing on the abilities of people in the population, including issues of health literacy, as well as supporting training for practice staff to enable them to work more effectively within current constraints. This might, for example, support staff in understanding how they can, without increasing their workload, find ways of helping patients to ‘feel known’.

Although this research took place before the COVID-19 pandemic, the understanding of access as human fit continues to be relevant. There were major changes to general practice appointments due to the pandemic,^47^ that is, remote consultation, which occurred out of necessity and without reference to patient preferences, and are likely to be associated with worse experiences of access for some groups, while potentially improving access for others. Further research can utilise the concept of human fit to understand the impacts of changes made, including on continuity of care. It could be used to support practices to think through which aspects of changes should be retained and which require modification to address potentially worsening inequalities.
